# Supplementary material for: Discovery of a lectin domain that regulates enzyme activity in mouse N-acetylglucosaminyltransferase-IVa (MGAT4A)
Source: Commun Biol. 2022 Jul 19;5:695. doi: 10.1038/s42003-022-03661-w (PMC9296478; doi:10.1038/s42003-022-03661-w)
Supplement: Supplementary file 2 — Supplementary Information [file 42003_2022_3661_MOESM2_ESM.pdf]

Supplementary Table 1

Primers used in this study

full length mGnT-IVa (pCR-Blunt II-TOPO) For	GAAATGAGATGAGGCTCCGAAATGG
full length mGnT-IVa (pCR-Blunt II-TOPO) Rev	GTTTCTAAGCAGATCAACTGGTGAC
full length mGnT-IVa (pcDNA6/myc-HisA) For	TGTGCGGCCGCGCCACCATGAGGCTCCGAAATGG
full length mGnT-IVa (pcDNA6/myc-HisA) Rev	AGCCTCGAGACTGGTGACTTTTTTAATAT
His-mGnT-IVa (60-526) For	CGGAGCTAAACACCATTGTC
His-mGnT-IVa (60-526) Rev	GTGCTCGAGTCTAAGCAGATCAACTGGTG
His-mGnT-IVa (78-526) For	ATGGAAATAATACCATAAAG
His-mGnT-IVa (78-526) Rev	GTGCTCGAGTCTAAGCAGATCAACTGGTG
His-mGnT-IVa (104-526) For	TGCCTCATCTATTGCAAAAT
His-mGnT-IVa (104-526) Rev	GTGCTCGAGTCTAAGCAGATCAACTGGTG
His-mGnT-IVa (60-361) For	CGGAGCTAAACACCATTGTC
His-mGnT-IVa (60-361) Rev	TTTCTCGAGTCACCCCGACAGAGACGAGTGTA
His-mGnT-IVΔ4 Fragment1 For	TCACTGCAGCTCGGAGCTAAACACCATTG
His-mGnT-IVΔ4 Fragment1 Rev	CGACAGAGACGAGTGTAGGC
His-mGnT-IVΔ4 Fragment2 For	AAACTCACGGATAAAGATTACA
His-mGnT-IVΔ4 Fragment2 Rev	GTGCTCGAGTCTAAGCAGATCAACTGGTG
His-mGnT-IVΔ9 Fragment1 For	TCACTGCAGCTCGGAGCTAAACACCATTG
His-mGnT-IVΔ9 Fragment1 Rev	CGACAGAGACGAGTGTAGGC
His-mGnT-IVΔ9 Fragment2 For	GATTACATGAAGCCATTGCT
His-mGnT-IVΔ9 Fragment2 Rev	GTGCTCGAGTCTAAGCAGATCAACTGGTG
His-mGnT-IVΔ19 Fragment1 For	TCACTGCAGCTCGGAGCTAAACACCATTG
His-mGnT-IVΔ19 Fragment1 Rev	CGACAGAGACGAGTGTAGGC
His-mGnT-IVΔ19 Fragment2 For	CACGTGAACCCGCCTGCAGA
His-mGnT-IVΔ19 Fragment2 Rev	GTGCTCGAGTCTAAGCAGATCAACTGGTG
D445A For	CAATCAAGAGCACCCAGGAGCCATCCTGCTGAACACGACCG
D445A Rev	CGGTCGTGTTCAGCAGGATGGCTCCTGGGTGCTCTTGATTG
T227A For	GGAAAGAGTGAGATGGAGAGCCAAGCAAAACCTGGATTACT
T227A Rev	AGTAATCCAGGTTTTGCTTGGCTCTCCATCTCACTCTTTCC
GST-GnT-IVa lectin domain (382-526) For	CTGTTCCAGGGGCCCCTGGGAAACCCGCCTGCAGAGGTC
GST-GnT-IVa lectin domain (382-526) Rev	TCGACCCGGGAATTCCGGGGATCAACTGGTGACTTTTTTAATATG
GST-GnT-IVa lectin domain (382-526) vector For	TCCCCGGAATTCCCGGGTCGA
GST-GnT-IVa lectin domain (382-526) vector Rev	TCCCAGGGGCCCCTGGAACAG
MGAT4A KO guide 1 For	caccGAAGACGTTCTTTCAAAGCA
MGAT4A KO guide 1 Rev	aaacTGCTTTGAAAGAACGTCTTC
MGAT4A KO guide 2 For	caccGTGCAACAGTTCAAGCGTGT
MGAT4A KO guide 2 Rev	aaacACACGCTTGAACTGTTGCAC
MGAT4B KO guide 1 For	caccACGTTGTGGACGTTTACCAG
MGAT4B KO guide 1 Rev	aaacCTGGTAAACGTCCACAACGT
MGAT4B KO guide 2 For	caccCGGAGACGGCAATCGCACCT
MGAT4B KO guide 2 Rev	aaacAGGTGCGATTGCCGTCTCCG
Genotyping of MGAT4A For	ATATGAAGTGCTGGAGACTGCTCAC
Genotyping of MGAT4A Rev	TGTAGTCCCAGCTATTCAGGAGG
Genotyping of MGAT4B For	CCCCATGAGCATTAGTGTCAGTG
Genotyping of MGAT4B Rev	GCTGCAGACTGCTCTCCTTGG

Supplementary Table 2

MM/GBSA binding energies of the glycans in the β1-2GlcNAc and β1-4GlcNAc binding modes. The ± values are standard deviations of the binding energies calculated over 500 frames.

(kcal/mol)

LIGAND	β1–2GlcNAc	β1–4GlcNAc
#103*	–13.8 ± 8.3	NA
#105	–25.5 ± 6.9	–18.5 ± 4.5
#107	–25.6 ± 6.0	–21.1 ± 5.0
#108	–34.9 ± 5.9	–20.7 ± 4.4

*The β1-2 GlcNAc was grafted into the primary binding site but the glycan dissociated after ~200 ns of molecular dynamics. It lacks β1-4 GlcNAc, and therefore the alternate binding mode is not feasible.

Supplementary Figure 1

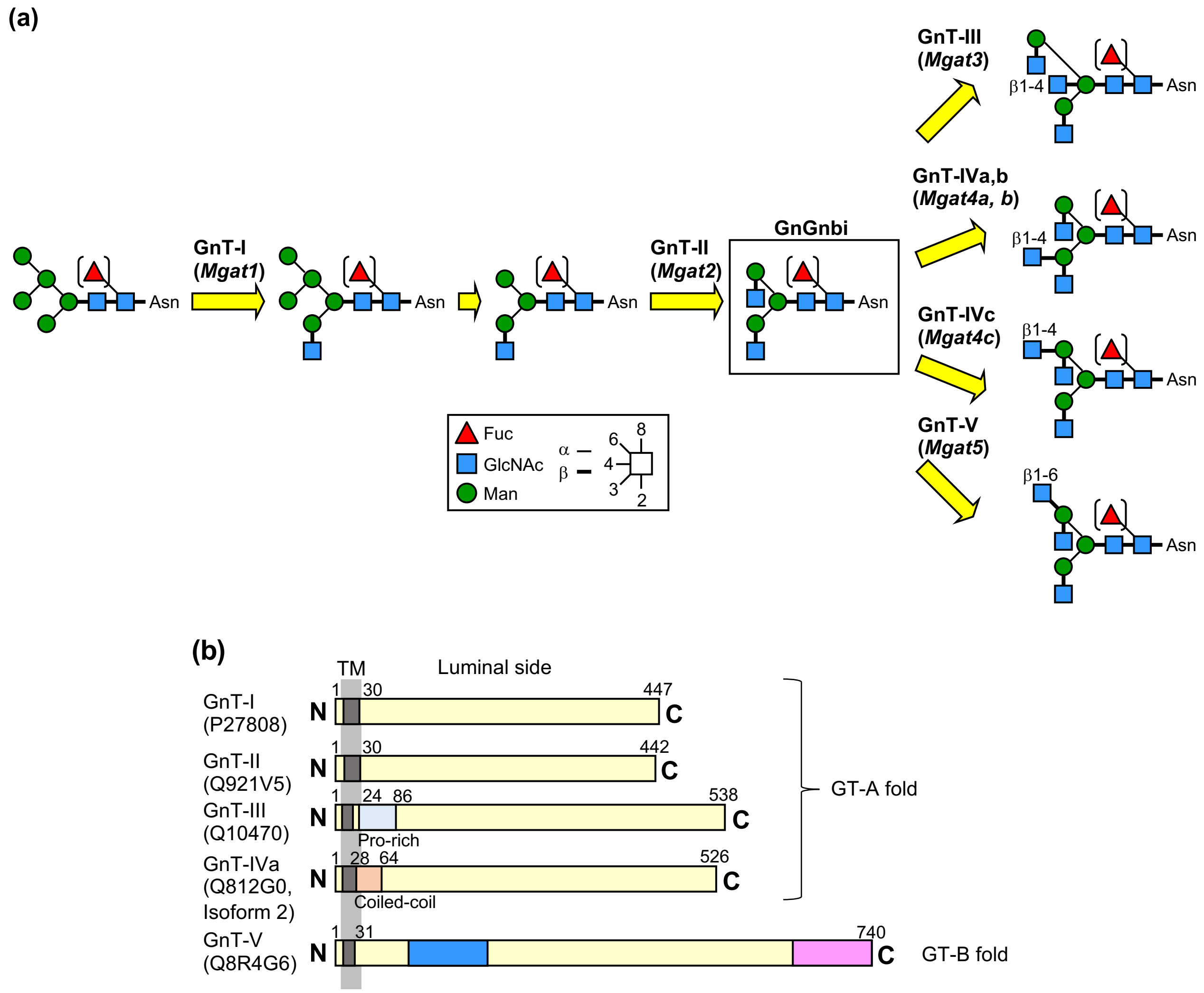

Supplementary Figure 1
(a) Schematic representation of *N*-glycan maturation. The biantennary *N*-glycan is modified to various types of triantennary glycan by GnT-III, IVa,b, IVc, and V. (b) Comparison of the domain architecture of the luminal regions of murine GnTs.

Supplementary Figure 2

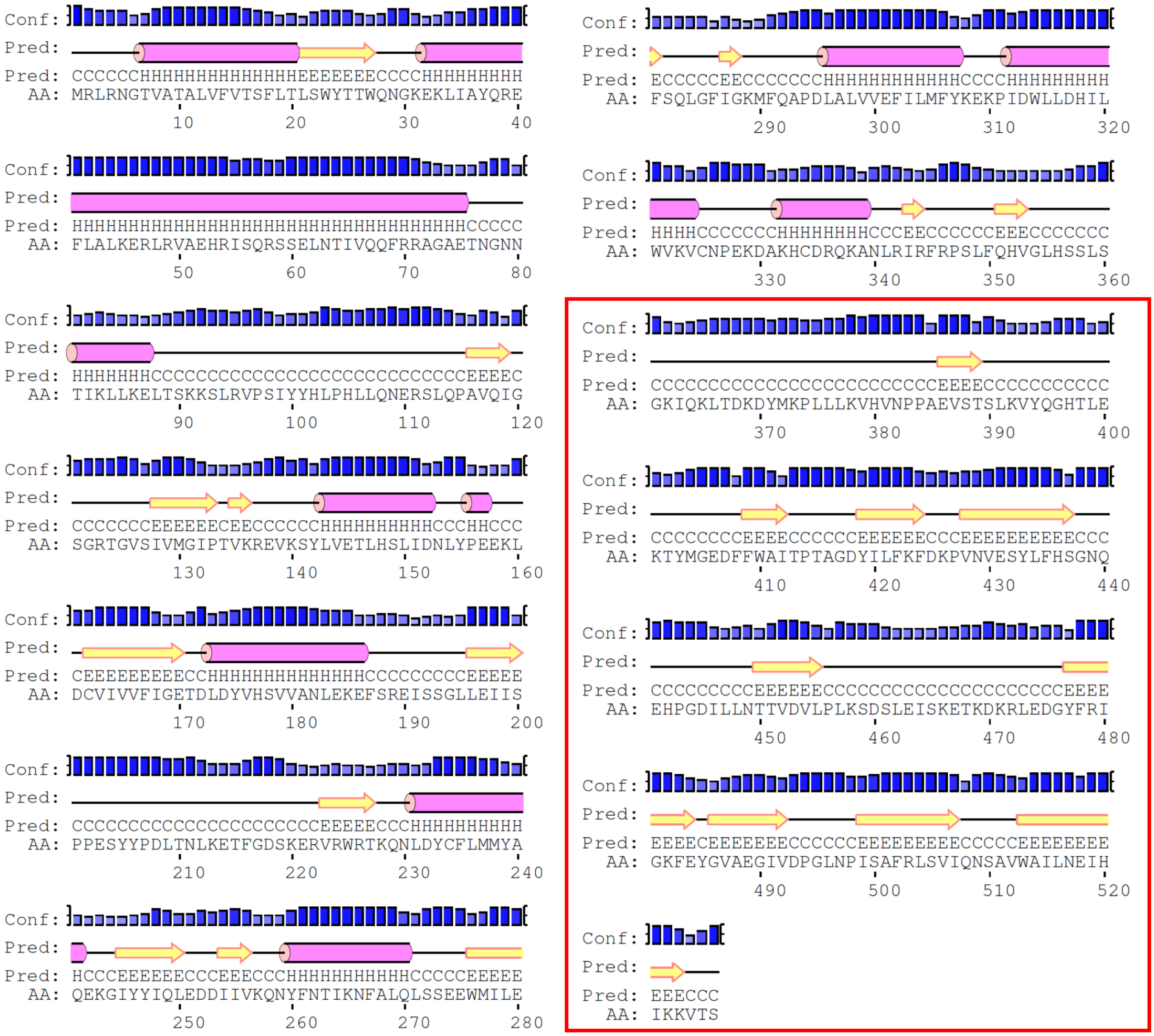

Supplementary Figure 2
Secondary structure prediction of mouse GnT-IVa depicted by PSIPRED server (69). The position of the lectin domain is indicated with a red box.

Supplementary Figure 3

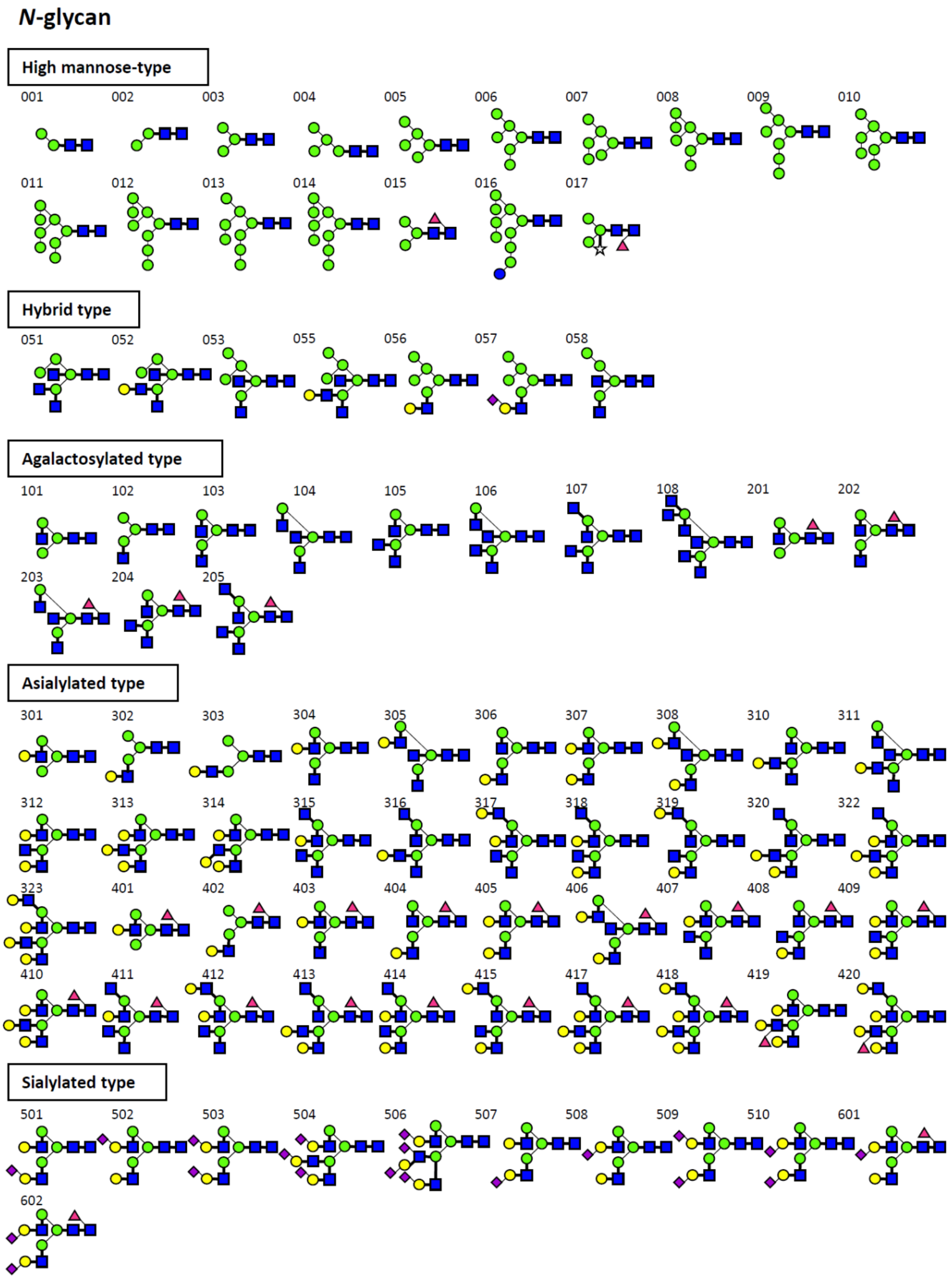

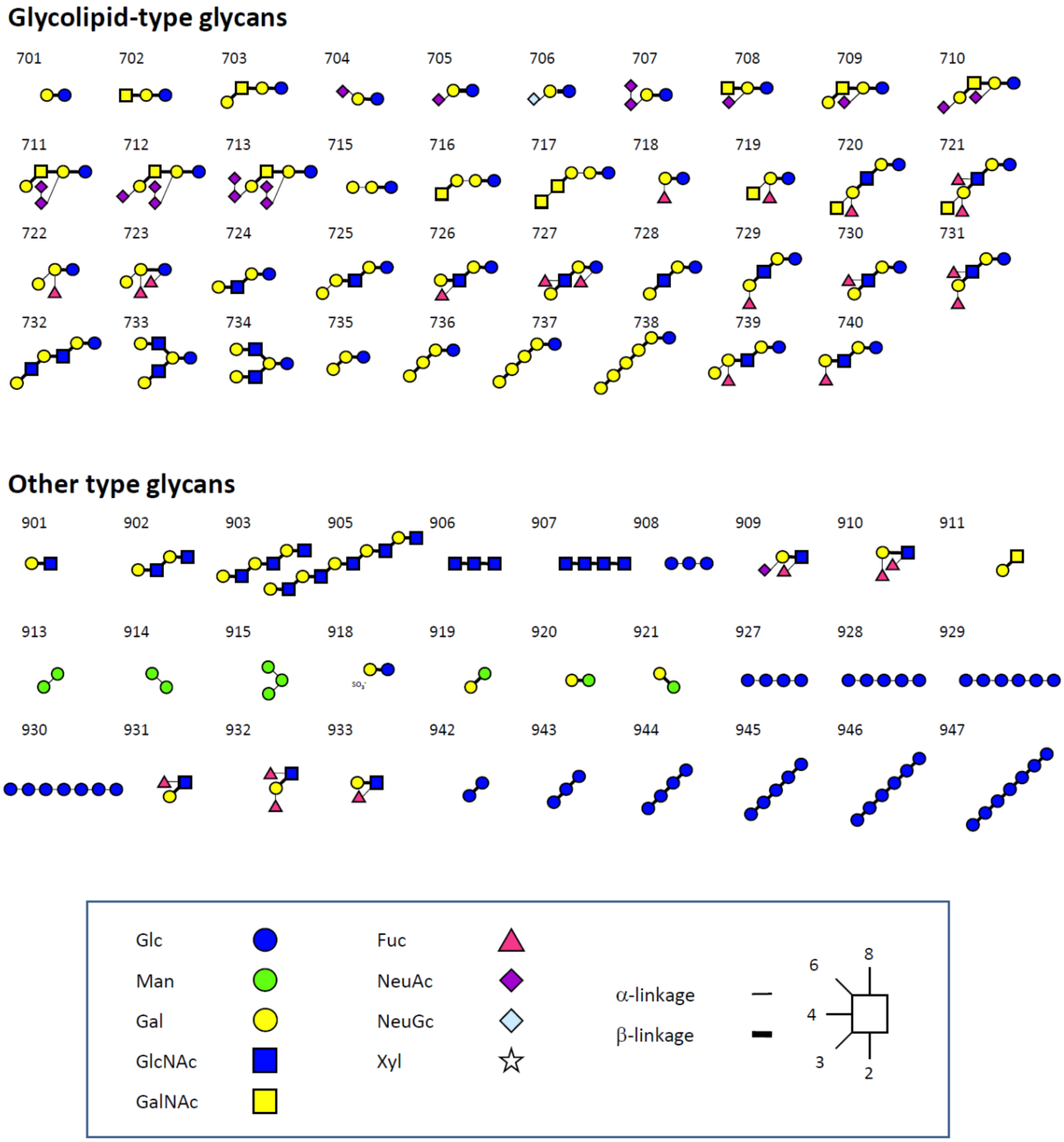

Supplementary Figure 3
Schematic representation of pyridylaminated (PA) sugars used in frontal affinity chromatography.

Supplementary Figure 4

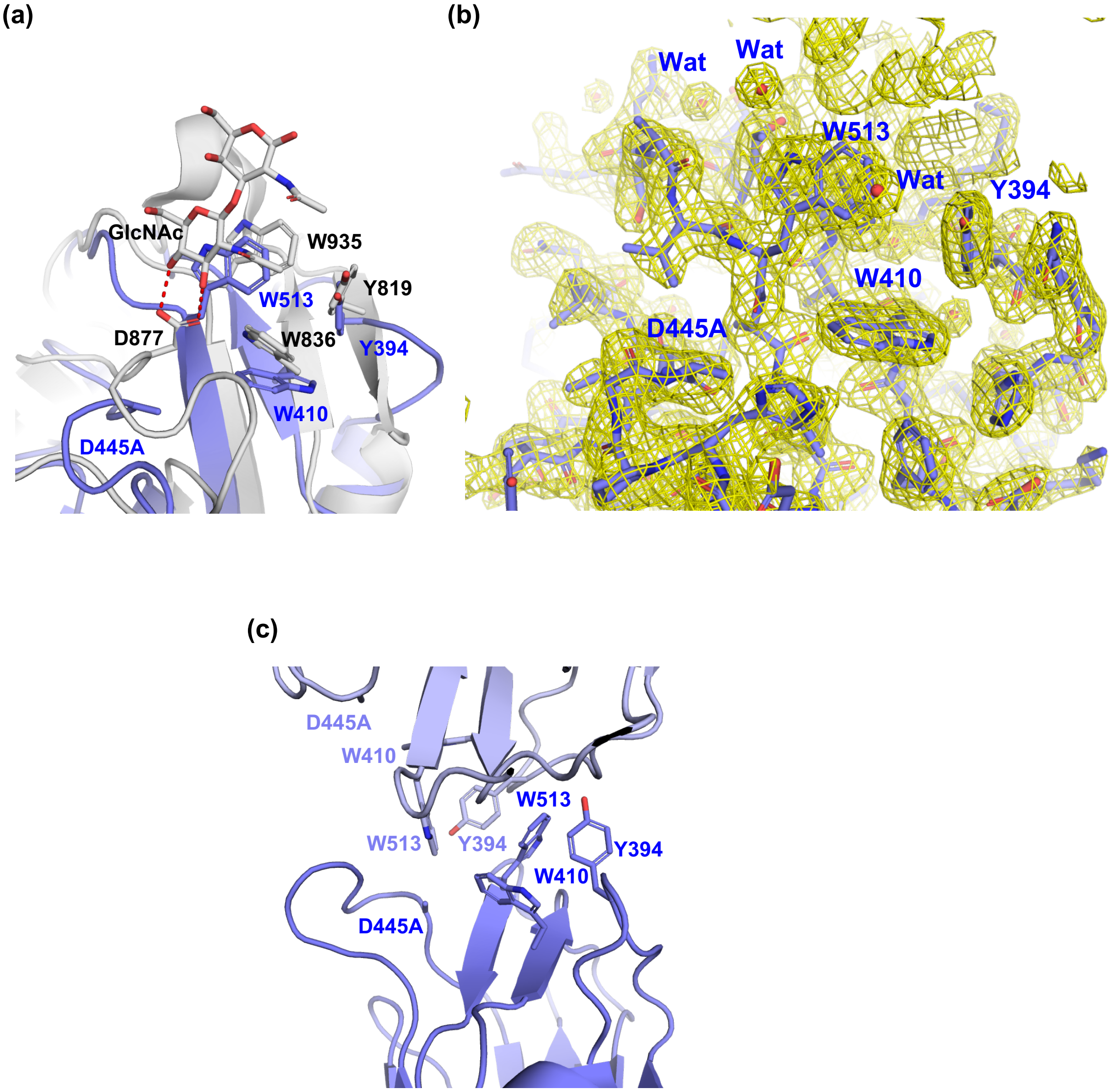

Supplementary Figure 4
(a) Structural superposition between GnT-IVa lectin domain (blue) and NagH-disaccharide complex (PDB code: 2W1U, white). This figure is depicted from the same view angle of Figure 3D. (b) The electron density map around D445A in GnT-IVa lectin domain. 2Fo-Fc map contoured at 1.5 σ level is shown in yellow mesh. (a) and (b) are depicted from same view angle. (c) Crystal packing of GnT-IVa lectin domain. Two molecules in the crystal are colored in blue and slate. The amino acid residues at the putative sugar binding sites are shown in stick model.

Supplementary Figure 5

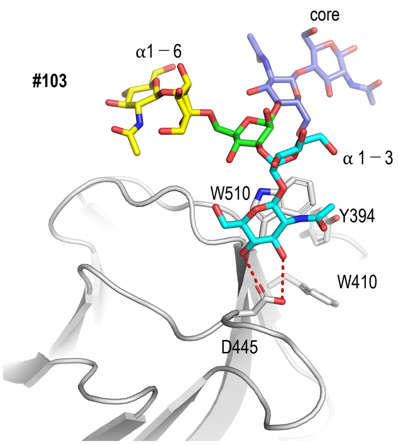

Supplementary Figure 5

Initial binding mode of glycan #103 after grafting over GlcNAc from NagH. The α1-3 arm (C cyan), α1-6 arm (C yellow) and chitobiose core (C blue) of the glycan have been colored differently. Red dashed lines show polar interactions between the glycan and D445. The glycan drifts away from the binding site and becomes unbound after ~200 ns of MD simulation.

Supplementary Figure 6

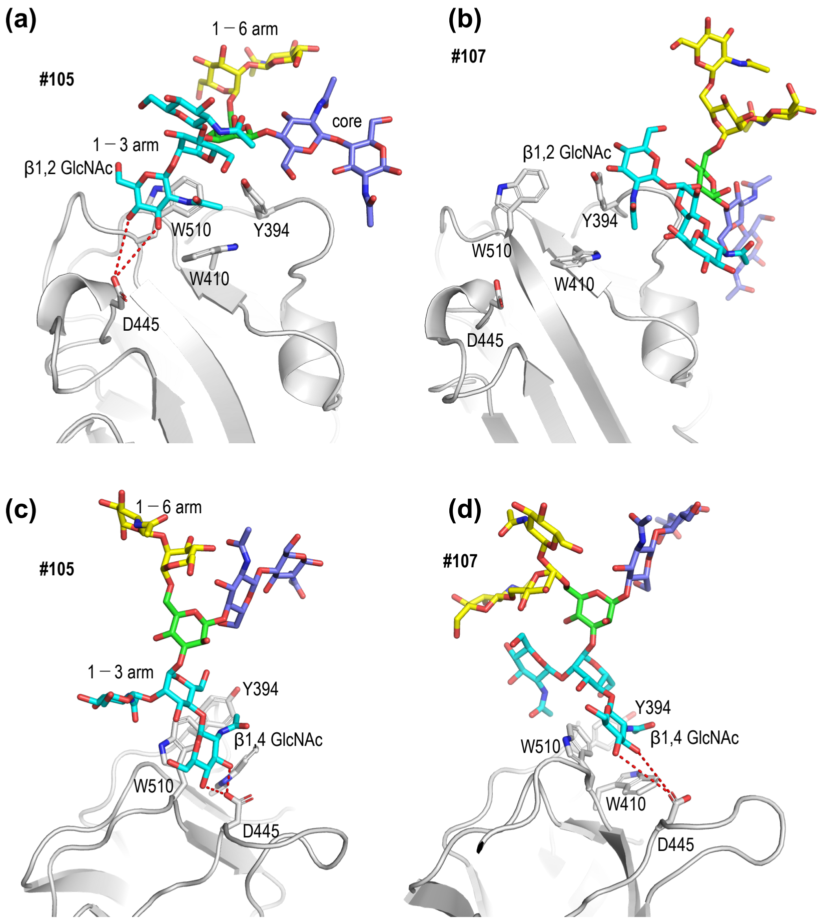

Supplementary Figure 6
Snapshots of binding mode of the glycans #105 and #107 after 500 ns molecular dynamics in both the β1-2GlcNAc (upper panel; a and b) and β1-4GlcNAc (lower panel; c and d) binding modes. The α1-3 arm (C cyan), α1-6 arm (C yellow) and chitobiose core (C blue) of the glycans have been colored differently. Red dashed lines show polar interactions between glycan and D445. Glycan #107 in the β1-2GlcNAc binding mode shifts from the known binding site but it continues to interact with neighboring binding site residues through the β1-2GlcNAc of the α1-3 arm.

Supplementary Figure 7

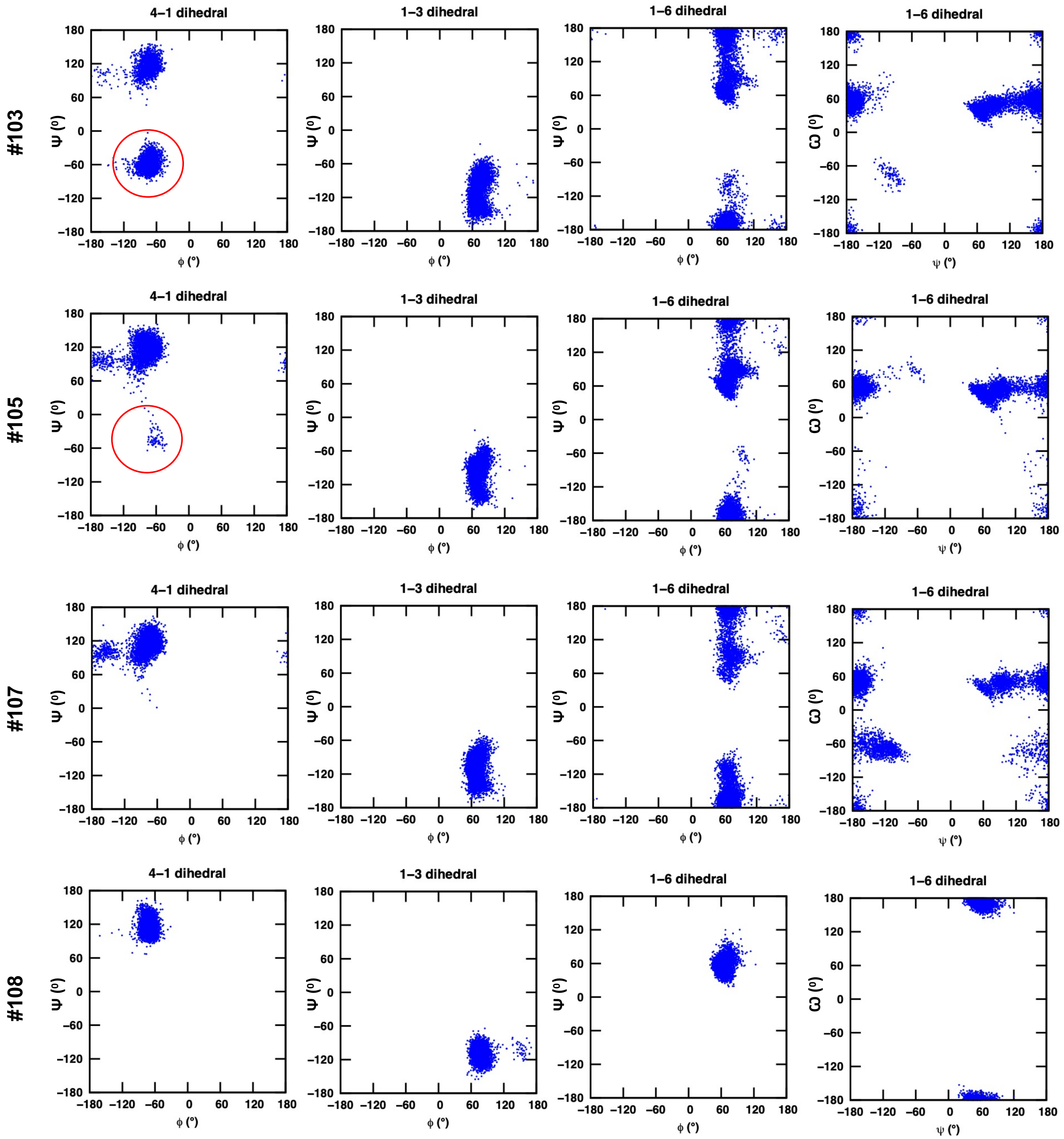

Supplementary Figure 7
The most relevant conformations of the *N*-glycan branches are characterized through the glycosidic ϕ, ψ and w angles. For a 1-x glycosidic linkage (where x is 2, 4 or 6 here), the glycosidic dihedral angles, ϕ and ψ, are defined as O_5-C_1-O_X-C_X and C_1-O_X- C_X-C_{X-1}. The w angle present only in 1-6 glycosidic linkage is defined as O_6- C_6-C_5-C_4.

Supplementary Figure 8

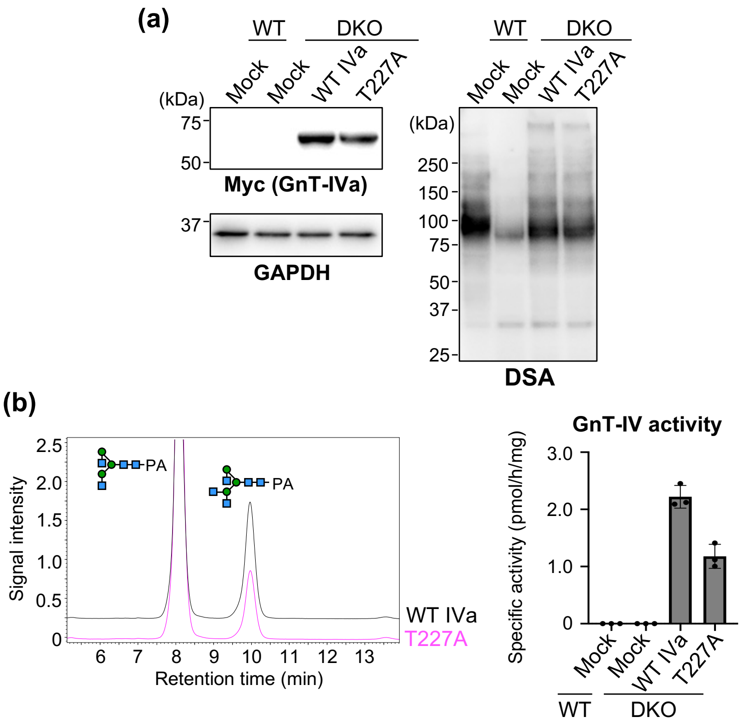

Supplementary Figure 8

(a) Proteins from mock-treated HEK293 wild-type and *MGAT4A*/*MGAT4B* double-knockout (DKO) HEK293 cells transfected with empty vector (mock), WT GnT-IVa-myc vector, or GnT-IVa T227A-myc vector were subjected to SDS-PAGE and blotted with anti-myc antibody (upper left), anti-GAPDH antibody (lower left), or HRP-conjugated DSA (right). (b) Lysates of DKO cells transfected with a plasmid for expression of WT GnT-IVa or T227A mutant were reacted with GnGnbi-PA and analyzed by HPLC (left). The GnT-IV specific activities of the lysates are shown (n = 3) (right). The graph shows mean ± S.D.

Supplementary Figure 9

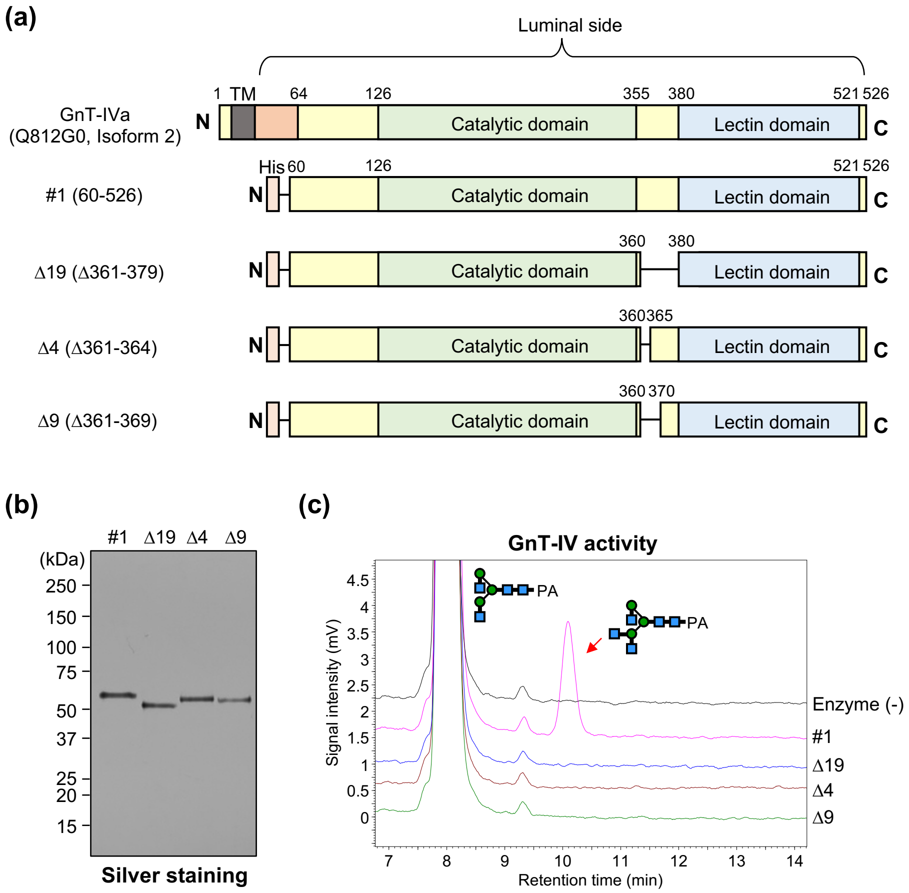

Supplementary Figure 9
(a) Constructs used for soluble GnT-IVa enzyme (#1) and the deletion mutants lacking a part of the linker region between the catalytic and C-terminal regions. (b) Purity of the enzymes prepared from COS7 media was checked by SDS-PAGE and subsequent silver staining. (c) Enzyme activity of the purified proteins was measured by incubation with the PA-labeled acceptor sugar and analyzed by HPLC.

Supplementary Figure 10

```
MGT4A_MOUSE     M R L R N G T V A T A L V F V T S - F L T L S W Y T T W Q N G K - E K L I A Y Q R E F L A L K E R L
MGT4B_MOUSE     M R L R N G T F L T L L L F C L C A F L S L S W Y A A L S G Q K G D V V D I Y Q R E F L A L R D R L
MGT4C_MOUSE     - - - - - - - - - - - - - - - M L K F Y Q M K Y I F Q I L D K M R C L R K R S T V S F L G V L V V F
MGT4D_MOUSE     M K A K N V N L L F A F V A V L L F G F S C F C I S R M N Q T N - N Q L I N C R N H V L E F K E I M
                                                                                                    . *   .       :

MGT4A_MOUSE     R V A E H R I S Q R S S E L N T I V Q Q F R R A G A E T N G S K - - - - - T A L S T I S D N T I K L
MGT4B_MOUSE     H A A E Q E S L K R S K E L N L V L E E I K R A V S E R Q A L R D G E G N R T W G R L T E D P R L K
MGT4C_MOUSE     L L F M N L Y I E D S Y V L E G D K Q L I R E T S T H Q L N S E - - - - - - - - - - - - - - - - - -
MGT4D_MOUSE     L R L K N K S E N H H Q D L M Q V L Y Q M K R K A A H T T R S S - - - - - - - G N F L E K K G S I L
                        :       :         *             : : .     : .

MGT4A_MOUSE     L K E L T S K K S L R V P S I Y Y H L P H L L Q N E R S L Q P A V Q I G S G R T G V S I V M G I P T
MGT4B_MOUSE     P W N V S H R H V L H L P T V F H H L P H L L A K E S S L Q P A V R V G Q G R T G V S V V M G I P S
MGT4C_MOUSE     - - - - - - - - - - R Y V H T F K D L S N F S G T I N V T Y R Y L A A T P L Q R K R Y L T I G L S S
MGT4D_MOUSE     S Q H E T L P N - - Q F E V L K Y F L P H L R T A G - K L Y P A I A T S K G R A G V S F A L G I S T
                                    :               * . : :                     :           :         . . : * : . :

MGT4A_MOUSE     V K R E V K S Y L V E T L H S L I D N L Y P E E K L D C V I V V F I G E T D L D Y V H S V V A N L E
MGT4B_MOUSE     V R R E V H S Y L T D T L H S L I S E L S P Q E K E D S V I V V L I A E T D P Q Y T S A V T E N I K
MGT4C_MOUSE     V K R K K G N Y L L D T I K S I F E Q S S Y E E L K E I S V V V H L A D F N S S W R D A M V Q D I T
MGT4D_MOUSE     I N R G N H T Y L K Q T L T S V L S R M T P E E E E D S V V I V S V A D T D E S Y L K S V V R M V K
                : . *       . * *   : * :   * : : . .       : *     :     : : *   : . :   :   . :     : : .     :

MGT4A_MOUSE     K E F S R E I S S G L L E I I S P P E S Y Y P D L T N L K E T F G D S K E R V R W R T K Q N L D Y C
MGT4B_MOUSE     A L F P T E I H S G L L E V I S P S P H F Y P D F S R L R E S F G D P K E R V R W R T K Q N L D Y C
MGT4C_MOUSE     Q K F A H H I I A G R L M V I H A P E E Y Y P V L D G L K R N Y N D P E D R V R F R S K Q N V D Y A
MGT4D_MOUSE     T K F R K Q V Q S G V L E V I S I P T L F Y P Q T L L D K K T K T D S E S - - - W Q I K Q V L D F C
                    *     . :   : *   *   : *     .     : * *           : . .     * . : .       : :   * *   : * : .

MGT4A_MOUSE     F L M M Y A Q E K G I Y Y I Q L E D D I I V K Q N Y F N T I K N F A L Q L S S E E W M I L E F S Q L
MGT4B_MOUSE     F L M M Y A Q S K G I Y Y V Q L E D D I V A K P N Y L S T M K N F A L Q Q P S E D W M I L E F S Q L
MGT4C_MOUSE     F L L N F C A N T S D Y Y V M L E D D V R C S R N F L T A I K K V I A S L E G T Y W V T L E F S K L
MGT4D_MOUSE     I L M L Y A Q P K A T Y Y L Q L E D D I V A K K M Y F T K M K D F V N S L T S K N W F F I E F S V L
                : * :   : .     . .   * * :   * * * * :     .     : : .   : * . .     .     .     * .   : * * *   *

MGT4A_MOUSE     G F I G K M F Q A P D L A L V V E F I L M F Y K E K P I D W L L D H I L W V K V C N P E K D A K H C
MGT4B_MOUSE     G F I G K M F K S L D L S L I V E F I L M F Y R D K P I D W L L D H I L W V K V C N P E K D A K H C
MGT4C_MOUSE     G Y I G K L Y H S H D L P R L A H F L L M F Y Q E M P C D W L L T H F R G L L A Q K N - - - - - - -
MGT4D_MOUSE     G F I G K L F R S K D L T D F V H F F L M F Y E T K P I D I L L D D I F L I R V C I S G E P V R S C
                * : * * * : : : :   * * .   . . . * : * * * * .     *   *   * *   . :     :   .

MGT4A_MOUSE     D R Q K A N L R I R F R P S L F Q H V G L H S S L S G K I Q K L T D K D Y M K P L L L K V H V N P P
MGT4B_MOUSE     D R Q K A N L R I R F K P S L F Q H V G T H S S L A G K I Q K L K D K D F G K H A L R K E H V N P P
MGT4C_MOUSE     - - - - - - - V I R F K P S L F Q H M G Y Y S S Y K G T E N K L K D D D F E E E S F D I P D N P P A
MGT4D_MOUSE     L Q R K K G F R I Q Y R P S L F Q H V G T Q S S F P G R E Q H L K D N Y Y - - - - - - - - - - - - -
                                * : : : * * * * * * : *     * *     *     : : * . * .   :

MGT4A_MOUSE     A E V S T S L K V Y Q G H T L E K T Y M G E D F F W A I T P T A G D Y I L F K F D K P V N V E S Y L
MGT4B_MOUSE     A E V S T S L K T Y Q H F T L E K A Y L R E D F F W A F T P A A G D F I R F R F F Q P L R L E R F F
MGT4C_MOUSE     S F Y T N M N V F E N Y E A S K A Y S S V D E Y F W G K S P S M G D T F V I V F E N P I T I K K I K
MGT4D_MOUSE     - - - - - - - - - - - - - - - - - - - - - - - - - - - - - - - - - - - - - - - - - - - - - - - - - -

MGT4A_MOUSE     F H S G N Q E H P G D I L L N T T V D V L P L K S D S L E I S K E T K D K - - - - - - - R L E D G Y
MGT4B_MOUSE     F R S G N I E H P E D K L F N T S V E V L P F D N P Q S E K E A L Q E G R S A T L R Y P R S P D G Y
MGT4C_MOUSE     V N T G T E D R Q N D I L Q H G A L D V G - - - - - - - - - - - - - - E K L I F S K Q I R Q C D T Y
MGT4D_MOUSE     - - - - - - - - - - - - - - - - - - - - - - - - - - - - - - - - - - - - - - - - - - - - - - - - - -

MGT4A_MOUSE     F R I G K F E Y G V A E G - - I V D P G L N P I S A F R L S V I Q N S A V W A I L N E I H I K K V T S
MGT4B_MOUSE     L Q I G S F Y K G V A E G - - E V D P A F G P L E A L R L S I Q T D S P V W V I L S E I F L K K A D -
MGT4C_MOUSE     L R L G E F K N G Y F E M S D V N Q K I P F D I H C M R I C V T K T Q K E W L I I R S I S I W T S - -
MGT4D_MOUSE     - - - - - - - - - - - - - - - - - - - - - - - - - - - - - - - - - - - - - - - - - - - - - - - - - - -
```

Supplementary Figure 10

Multiple sequence alignment of murine GnT-IVa–d. The accession numbers of protein sequences are as follows: Q812G0 (MGT4A_MOUSE), Q812F8 (MGT4B_MOUSE), Q9D306 (MGT4C_MOUSE), and Q9D4R2 (MGT4D_MOUSE). Amino acid residues that form the putative catalytic center are highlighted in red. Four amino acid residues (Y394, W410, D445, and W513 in IVa) at the sugar binding site are highlighted in yellow.

Supplementary Figure 11

Fig. 1f

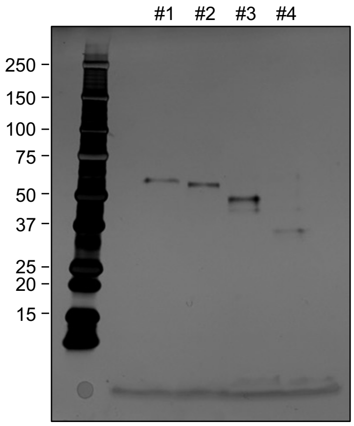

Fig. 2a

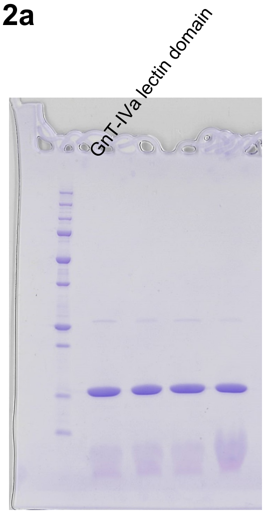

Fig. 3a

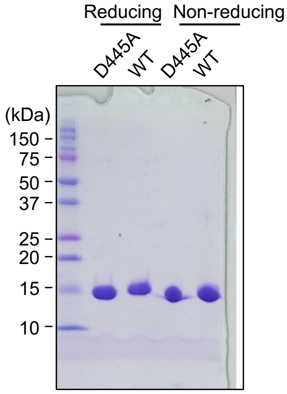

Fig. 5b

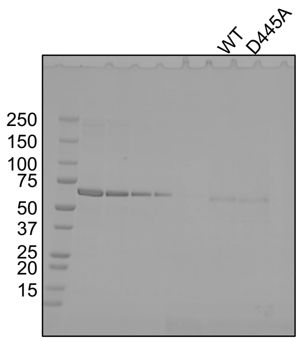

Fig. 5e

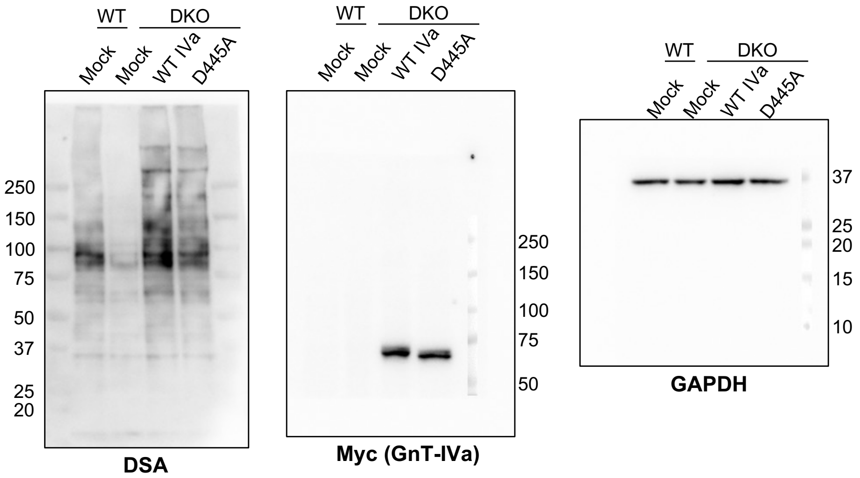

Fig. S8a

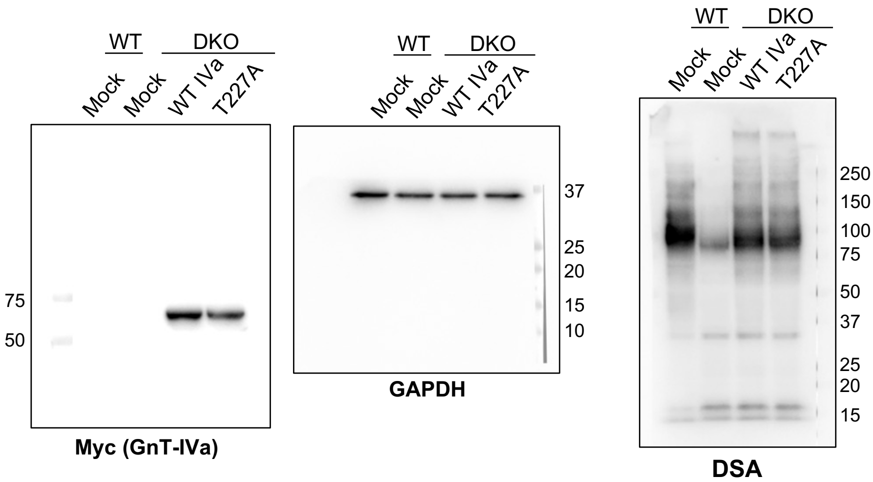

Fig. S9b

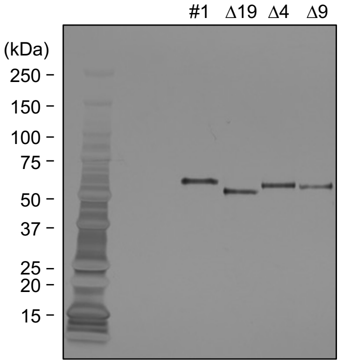

Supplementary Figure 11
Source data for all the gel/blots in this paper.
