# Supplementary material for: Discovery of a lectin domain that regulates enzyme activity in mouse N-acetylglucosaminyltransferase-IVa (MGAT4A)
Source: Commun Biol. 2022 Jul 19;5:695. doi: 10.1038/s42003-022-03661-w (PMC9296478; doi:10.1038/s42003-022-03661-w)
Supplement: Supplementary file 5 — Reporting Summary [file 42003_2022_3661_MOESM5_ESM.pdf]

nature portfolio

Corresponding author(s): Masamichi Nagae, Yasuhiko Kizuka

Last updated by author(s): Jun 8, 2022

# Reporting Summary

Nature Portfolio wishes to improve the reproducibility of the work that we publish. This form provides structure for consistency and transparency in reporting. For further information on Nature Portfolio policies, see our Editorial Policies and the Editorial Policy Checklist.

## Statistics

For all statistical analyses, confirm that the following items are present in the figure legend, table legend, main text, or Methods section.

| n/a | Confirmed | |
|---|---|---|
| ☐ | ☒ | The exact sample size (*n*) for each experimental group/condition, given as a discrete number and unit of measurement |
| ☐ | ☒ | A statement on whether measurements were taken from distinct samples or whether the same sample was measured repeatedly |
| ☐ | ☒ | The statistical test(s) used AND whether they are one- or two-sided<br>*Only common tests should be described solely by name; describe more complex techniques in the Methods section.* |
| ☒ | ☐ | A description of all covariates tested |
| ☒ | ☐ | A description of any assumptions or corrections, such as tests of normality and adjustment for multiple comparisons |
| ☐ | ☒ | A full description of the statistical parameters including central tendency (e.g. means) or other basic estimates (e.g. regression coefficient) AND variation (e.g. standard deviation) or associated estimates of uncertainty (e.g. confidence intervals) |
| ☐ | ☒ | For null hypothesis testing, the test statistic (e.g. *F*, *t*, *r*) with confidence intervals, effect sizes, degrees of freedom and *P* value noted<br>*Give P values as exact values whenever suitable.* |
| ☒ | ☐ | For Bayesian analysis, information on the choice of priors and Markov chain Monte Carlo settings |
| ☒ | ☐ | For hierarchical and complex designs, identification of the appropriate level for tests and full reporting of outcomes |
| ☒ | ☐ | Estimates of effect sizes (e.g. Cohen's *d*, Pearson's *r*), indicating how they were calculated |

*Our web collection on statistics for biologists contains articles on many of the points above.*

## Software and code

Policy information about availability of computer code

| | |
|---|---|
| Data collection | UGUIS, LCSolution, FACSChorus, Amber20 |
| Data analysis | XDS, POINTLESS, SCALA, CRANK2, COOT, REFMAC5, Phenix.refine, LCSolution, FlowJo software (10.8.0), Amber20, GraphPad Prism 8 software |

For manuscripts utilizing custom algorithms or software that are central to the research but not yet described in published literature, software must be made available to editors and reviewers. We strongly encourage code deposition in a community repository (e.g. GitHub). See the Nature Portfolio guidelines for submitting code & software for further information.

## Data

Policy information about availability of data

All manuscripts must include a data availability statement. This statement should provide the following information, where applicable:

- Accession codes, unique identifiers, or web links for publicly available datasets
- A description of any restrictions on data availability
- For clinical datasets or third party data, please ensure that the statement adheres to our policy

Structural factors and atomic coordinates of GnT-IVa lectin domain have been deposited on Protein Data Bank under the accession code of 7VMT.

# Field-specific reporting

Please select the one below that is the best fit for your research. If you are not sure, read the appropriate sections before making your selection.

☒ Life sciences ☐ Behavioural & social sciences ☐ Ecological, evolutionary & environmental sciences

For a reference copy of the document with all sections, see nature.com/documents/nr-reporting-summary-flat.pdf

# Life sciences study design

All studies must disclose on these points even when the disclosure is negative.

| | |
|---|---|
| Sample size | We initially performed three independent experiments and determined whether additional experiments were required considering the tendency. |
| Data exclusions | No data were excluded from this study. |
| Replication | All attempts to replication were successful. |
| Randomization | This is not relevant to this study because the paper does not include comparison studies among group. |
| Blinding | The investigators were not blinded during data collection. |

# Reporting for specific materials, systems and methods

We require information from authors about some types of materials, experimental systems and methods used in many studies. Here, indicate whether each material, system or method listed is relevant to your study. If you are not sure if a list item applies to your research, read the appropriate section before selecting a response.

## Materials & experimental systems

| n/a | Involved in the study | |
|---|---|---|
| ☐ | ☒ | Antibodies |
| ☐ | ☒ | Eukaryotic cell lines |
| ☒ | ☐ | Palaeontology and archaeology |
| ☒ | ☐ | Animals and other organisms |
| ☒ | ☐ | Human research participants |
| ☒ | ☐ | Clinical data |
| ☒ | ☐ | Dual use research of concern |

## Methods

| n/a | Involved in the study | |
|---|---|---|
| ☒ | ☐ | ChIP-seq |
| ☐ | ☒ | Flow cytometry |
| ☒ | ☐ | MRI-based neuroimaging |

## Antibodies

| | |
|---|---|
| Antibodies used | Anti-myc (05-724) and anti-GAPDH (MAB374) antibodies were purchased from Millipore. |
| Validation | Validation is shown in supplier webpage. |

## Eukaryotic cell lines

Policy information about cell lines

| | |
|---|---|
| Cell line source(s) | COS7 cells were purchased from RIKEN cell bank. HEK293 were obtained from the American Type Culture Collection. HEK293-MGAT4A/MGAT4B double-knockout (DKO) cells were generated by ourselves. |
| Authentication | None of the cell lines were authenticated. |
| Mycoplasma contamination | All cell lines were not tested for mycoplasma contaminination. |
| Commonly misidentified lines (See ICLAC register) | HEK cells were listed, but COS7 cells were not. |

# Flow Cytometry

## Plots

Confirm that:

☒ The axis labels state the marker and fluorochrome used (e.g. CD4-FITC).

☒ The axis scales are clearly visible. Include numbers along axes only for bottom left plot of group (a 'group' is an analysis of identical markers).

☒ All plots are contour plots with outliers or pseudocolor plots.

☒ A numerical value for number of cells or percentage (with statistics) is provided.

## Methodology

| | |
|---|---|
| Sample preparation | Cells were washed with PBS twice and collected using cell scrapers, followed by centrifugation at 1,400 x g for 3 min. The cells were washed with FACS buffer (1% BSA, 0.1% NaN3, in PBS) once and stained with fluorescently-labeled lectin in FACS buffer on ice for 15 min. |
| Instrument | FACS Melody cell sorter (BD) |
| Software | Data were collected by FACSChorus software and analyzed using FlowJo software (10.8.0). |
| Cell population abundance | We collected 30,000 cells for each sample in which more than 50% was analyzed as viable cells. |
| Gating strategy | We used cell lines and gating of different populations does not apply to this study. |

☐ Tick this box to confirm that a figure exemplifying the gating strategy is provided in the Supplementary Information.
